# Bilateral Tolosa-Hunt syndrome mimicking pituitary adenoma

**DOI:** 10.1007/s12020-017-1422-2

**Published:** 2017-10-14

**Authors:** Renata Świątkowska-Stodulska, Dominik Stodulski, Anna Babińska, Maciej Piskunowicz, Krzysztof Sworczak

**Affiliations:** 1 Department of Endocrinology and Internal Medicine, Medical University of Gdańsk, Gdańsk, Poland; 2 Department of Otolaryngology, Medical University of Gdańsk, Gdańsk, Poland; 3 Department of Radiology, Medical University of Gdańsk, Gdańsk, Poland

**Keywords:** Tolosa-Hunt syndrome, Painful ophthalmoplegia, Pituitary adenoma

## Abstract

The authors report a rare case of bilateral Tolosa-Hunt syndrome, which occurred in a 80-year-old female and remitted spontaneously. Inflammatory lesions were found not only in typical locations, i.e. superior orbital fissures and cavernous sinuses, but also in the pituitary; these imitated gland’s macroadenoma in imaging studies.

## Introduction

Tolosa-Hunt syndrome is a rare cause of ophthalmoplegia due to a chronic unspecific inflammation that involves the cavernous sinus and/or superior orbital fissure. Clinically, the syndrome is characterized by strong orbital and retrobulbar pain, accompanied by oculomotor (III), trochlear (IV), and/or abducens (VI) nerve paresis of a varying degree. Disorders of sensation may also be present in the area innervated by the ocular branch of the trigeminal nerve (V1) [1, 2]. Lesions are usually unilateral; so far, only few bilateral Tolosa-Hunt syndrome cases have been reported [1, 3, 4].

The syndrome was first described in 1954 by Eduardo Tolosa in a patient with unilateral, recurrent, painful ophthalmoplegia involving cranial nerves III, IV, VI, as well as V1 [5]. For many years the diagnosis had been made by excluding other possible causes. Implementation of magnetic resonance imaging (MRI) facilitated identification of the inflammatory infiltrate, which is typically located in the superior orbital fissure or cavernous sinus, thus narrowing down possible differential diagnoses [6–12].

According to the 3rd edition of International Classification of Headache Disorders (ICHD-3 beta version) diagnostic criteria of Tolosa-Hunt syndrome are: unilateral headache that “has preceded paresis of the IIIrd, IVth and/or VIth nerves by ≤2 weeks, or developed with it”, and “is localized around the ipsilateral brow and eye”, and both of the following: “granulomatous inflammation of the cavernous sinus, superior orbital fissure or orbit demonstrated by MRI or biopsy” and “paresis of one or more of the ipsilateral IIIrd, IVth and/or VIth cranial nerves”, with no better ICHD-3 diagnosis that accounts for it [13–15].

Similar symptoms may be present due to: proliferative processes of the pituitary gland (in particular pituitary adenoma apoplexy); in patients with vascular disorders, such as cavernous sinus thrombosis, carotid-cavernous fistula, internal carotid artery aneurysm, vascular inflammation; proliferative processes of the orbit (neoplastic tumors, sarcoidosis, Wegener’s granulomatosis, eosinophilic granuloma); severe thyroid orbitopathy; and infectious diseases (bacterial sinusitis or meningitis, fungal, viral, and mycobacterial inflammation) [1–4, 6, 8].

We report a case of an 80-year-old female patient with symptoms of bilateral Tolosa-Hunt syndrome that remitted spontaneously and completely.

## Case report

Ms. W. T, an 80-year-old female patient, was consulted by an endocrinologist due to double vision with accompanying retro-orbital pain, particularly intense on the right side. These symptoms lasted for approximately 6–8 weeks prior to presentation and aggravated gradually. Eye symptoms were preceded by a headache that initially involved the right orbit; after several days similar symptoms developed in the left eye, but were much milder. Patient’s medical history included well-controlled hypertension, hypercholesterolemia treated with a statin; there was no history of other diseases or past head trauma.

The physical examination revealed bilateral swelling of the eyelids, conjunctival erythema and edema, as well as signs of right abducens nerve palsy: convergent strabismus of the right eye, lack of orbital movement toward the temple, diplopia that escalated while gazing toward the right side. The conjunctival sac was free of discharge. There were no signs of an endocrinopathy.

**Fig. 1:**
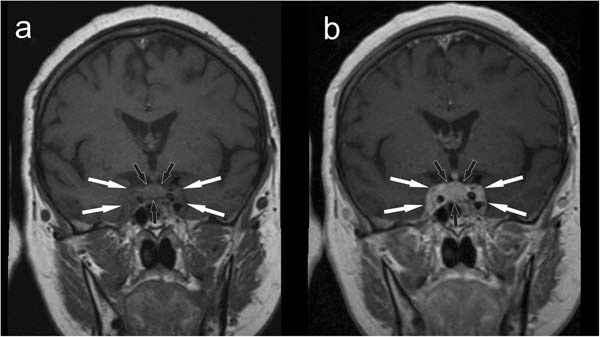
Initial MRI scan, coronal plane, before (**a**) and after (**b**) contrast medium administration. The pathological infiltrate involving the pituitary is marked with black arrows. White arrows mark the extent of the infiltrate, which involves both cavernous sinuses

**Fig. 2:**
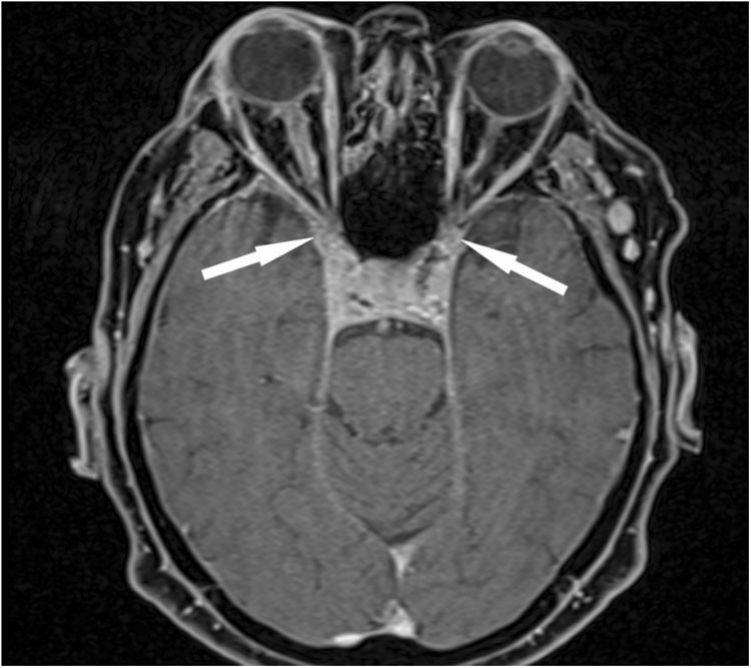
Initial MRI scan, axial plane, after contrast medium administration. White arrows indicate the infiltration that encompasses superior orbital fissures

After laryngological and ophthalmological consultations, which confirmed right abducens nerve paralysis and normal visual field, the patient was referred for an MRI of the pituitary gland and the orbits. An intrasellar, 11 × 13 × 13-mm tumor was revealed within the pituitary gland, whose features indicated a pituitary adenoma (Fig. 1). The lesion did not infiltrate the optic chiasm, it involved both cavernous sinuses but was much more marked in the right one. The infiltrate also involved both superior orbital fissures (Fig. 2). Moreover, mild segmental thickening of the right internal carotid artery was visible. Based on radiological features, a suspicion was raised of a concomitant pituitary adenoma and inflammatory infiltration in the course of Tolosa-Hunt syndrome.

Due to suspected sella turcica infiltration, the patient was admitted to the Department of Endocrinology and Internal Medicine of the Medical University of Gdańsk to perform a full hormonal work-up. On admission to the Department double vision was still present, however, partial remission of eyelid and conjunctival edema was observed, as was improved mobility of the right eyeball. In the neurological exam VIth nerve palsy was present without other abnormalities. Orbital pressure was normal, and exophthalmos was not present (exophthalmometric measurement result was 19 mm in the right eye, and 18 mm in the left), which was assessed by a consulting ophthalmologist. In a second MRI scan, performed 6 weeks after the previous one, enlargement of the pituitary persisted, there was a modest regression of the cavernous sinuses infiltration, particularly the right one, and lesions of the right internal carotid artery receded.

Hormonal tests showed normal function of the anterior and posterior lobe of the pituitary gland (serum concentrations: TSH 1.57 uU/ml; ACTH 12.6 pg/ml; morning cortisol 295 nmol/l; evening cortisol 84 nmol/l, PRL 266 mU/l, LH 34.21 U/l, FSH 81.66 IU/l, IGF-1- 144.8 ng/ml, GH 0.91 ng/ml; urine specific gravity 1.015 g/l). Complete blood count, biochemical, as well as inflammatory markers’ concentrations were unremarkable (WBC 5100/µl, CRP 4.91 mg/l, ESR 20 mm/h). Diabetes mellitus, thyroid disorders including an autoimmune disease (anti-TSH receptor, anti-TPO, anti-TG antibodies), and Lyme disease (IgM < 6.0 AU/ml, IgG < 5.0 AU/ml) were excluded. No laboratory indications of a connective tissue disease (including vasculitis) were found (anti-nuclear ANA-Hep 2, anti-dsDNA, anti-Sm, ANCA, anti-endothelial antibodies were negative). In a chest CT scan no pathologies were revealed (apart from an area of fibrosis in the posterior phrenicocostal sinus).

Subsequently, the patient was consulted by a neurosurgeon: in light of gradual remission of clinical symptoms, she was qualified neither for pituitary surgery, nor biopsy of the lesions. The decision was made to closely monitor the patient in the endocrine and neurological outpatient clinics.

Two months later, in a third MRI scan considerable regression of the infiltrate of the pituitary, cavernous sinuses, and superior orbital fissures (from 7 to 3–4 mm) was revealed. The pituitary height decreased from 11 to 9 mm, and the gland presented normal anterior and posterior lobe differentiation. Uptake of contrast medium was not homogenous in the anterior lobe, but there were no clearly visible lesions. There was residual infiltration in the upper part of the right and left cavernous sinuses: 1–2 and 2 mm, respectively. Radiological remission was accompanied by further clinical improvement. Taking into account the clinical course, exclusion of other causes of patient’s symptoms, localization as well as character of radiologic lesions, the patient was diagnosed with Tolosa-Hunt syndrome. The diagnosis was made based on ICHD criteria: asymmetric orbital pain, associated with ipsilateral paresis of the VIth cranial nerve, caused by inflammation in the cavernous sinus and superior orbital fissure. Eye symptoms were preceded by right-sided headache. The inflammatory process that involved cavernous sinuses and superior orbital fissures was confirmed in MRI scans. Diagnostic work-up ruled out other causes of painful ophthalmoplegia.

Glucocorticoids were not implemented since clinical symptoms receded. Eight weeks following the third MRI scan, symptoms reported by the patient (including diplopia) were no longer present. Physical examination showed full mobility of eyeballs, without edema or chemosis.

**Fig. 3:**
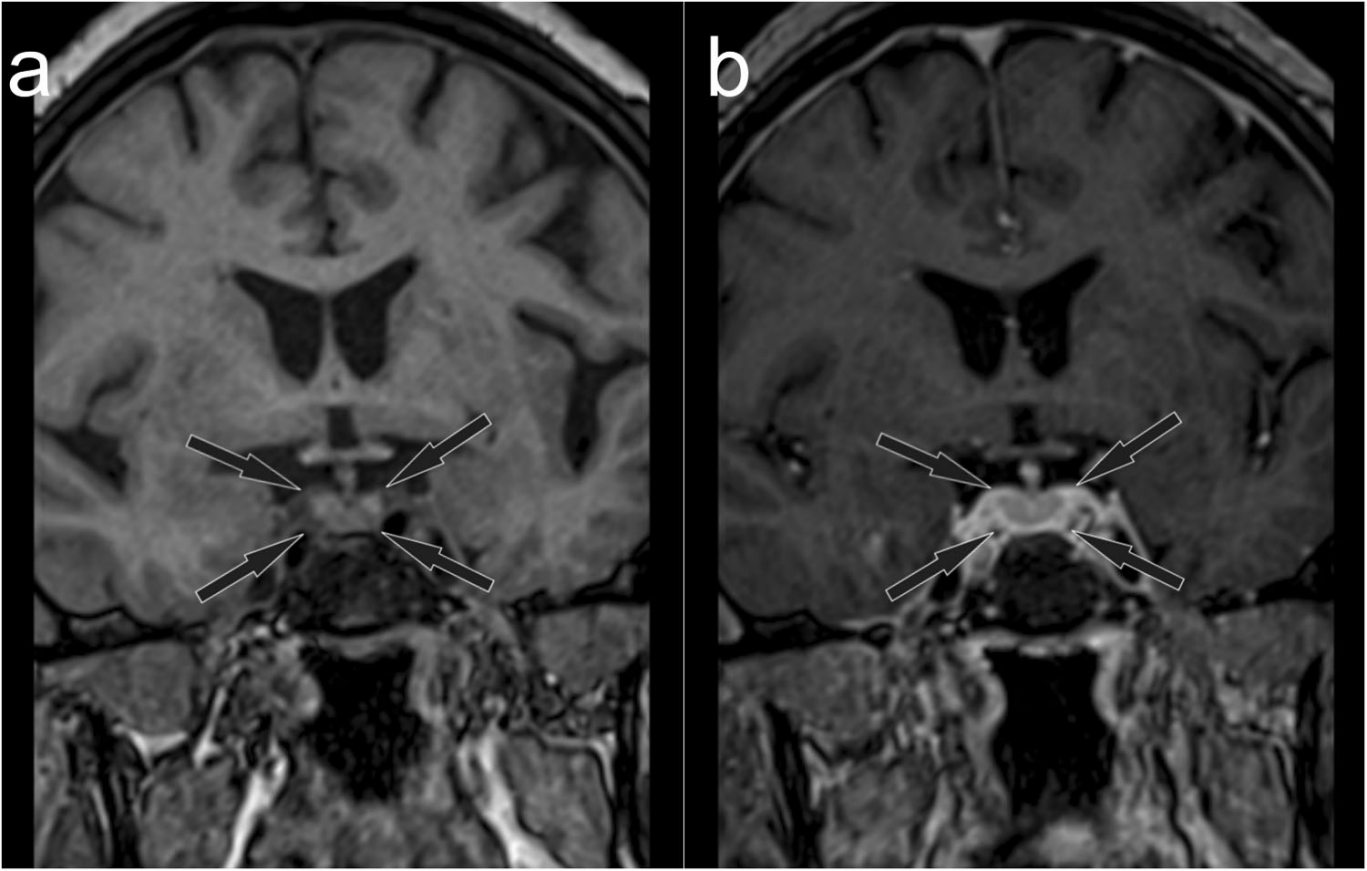
Follow-up MRI scan, coronal plane, before (**a**) and after (**b**) contrast medium administration. Normal enhancement of the pituitary gland (marked with black arrows)

Complete remission of the lesion described previously was revealed by MRI 6 months later: the pituitary height was 7 mm, the gland was clearly divided into two lobes, uptake of contrast medium was homogenous, and there were no focal lesions within the pituitary or neighboring tissues (Fig. 3).

The patient has been in good health, and has been followed-up in the outpatient endocrine clinic of the University Clinical Center in Gdańsk. The syndrome has not recurred in an almost 4-year observation.

## Discussion

Tolosa-Hunt syndrome is a rare cause of paresis or paralysis of oculomotor muscles, accompanied by hemicrania, pain of the peri- and retrobulbar area of the affected eye socket. The inflammatory infiltration usually involves the cavernous sinus and superior orbital fissure; it leads to ophthalmoplegia. Typically, the disease begins with a headache and pain of the retrobulbar area, followed by ophthalmoplegia of varying degree, ptosis, double vision, and—in the case of massive involvement of the cavernous sinus or orbit—also by chemosis [1–4, 6–8]. Severity of the symptoms depends on the location and extent of infiltrative lesions.

The etiology of the syndrome is unknown. While neurosurgical intervention is currently not recommended, in cases where samples were obtained for histopathological examination, granulomatous tissue with lymphocytes, plasmocytes, and proliferating fibroblasts was revealed. The prevalence of Tolosa-Hunt syndrome is approximately 1–2 cases per million, with an equal incidence among both sexes [6]. The disease may be recurrent. So far, patients with recurrences were significantly younger during their first episode compared to others [8].

Currently, the diagnosis is based on typical clinical and radiological (MRI) features; thus, a neurosurgical intervention and biopsy of lesions in a problematic region may be avoided [8, 12–17]. The diagnosis can be made, if painful ophthalmoplegia coexists with involvement of a characteristic area in a MRI scan. Taking into account the localization of infiltrative lesions, it is not recommended to obtain samples for histopathological examination. Therefore, MRI is key in diagnosing the syndrome. It has to be underlined, however, that Tolosa-Hunt syndrome cases without evident lesions in MRI scans were also reported in literature [15]. It is thought that at very early stages of the disease, in the period preceding cranial nerve palsy, this imaging study may not show typical infiltrative lesions. In such cases follow-up high resolution, fat-suppressed, contrast-enhanced MRI seems to be essential [15].

Paresis of the oculomotor nerve is more common than that of abducens or trochlear [18]. According to current criteria, lesions in the Tolosa-Hunt syndrome are unilateral. In our patient the infiltrate involved the superior orbital fissures and cavernous sinuses but was bilateral (more pronounced on the right). The infiltrate involved not only typical localization, but also the pituitary gland and it imitated an adenoma. Normal pituitary function despite a massive infiltration indicates that the inflammation of the gland is not a primary process. All other possible causes of lesions were excluded, both local and systemic. So far, only several cases of atypical, bilateral Tolosa-Hunt syndrome have been reported [1, 3, 4]. Classic treatment involves glucocorticoid administration—initially in immunosuppressive doses, with gradual dose reduction; the therapy lasts 4–6 weeks. Pain usually recedes within 72 h after the initial dose of the steroid, whereas nerve paralysis may persist for several weeks [1, 2, 4, 8, 14–16]. In a vast majority of reported cases, pain receded in the first 5 days, while signs of the oculomotor nerve palsy persisted up to 60 days after steroid treatment [15]. Patients who experience rapid improvement of orbit mobility were significantly younger that those without evident regression [8]. In our patient, immunosuppressive treatment was not implemented since on admission to the Department of Endocrinology symptoms abated evidently, which was confirmed by MRI. During the hospitalization further spontaneous regression was observed, i.e. absence of pain and improved mobility of the right orbit. For this reason standard glucocorticoid treatment, which might have led to adverse effects (especially in an elderly patient), was not initiated. The patient was carefully monitored, and steroids could have been implemented if symptoms had recurred.

The course of the disease in our patient supports current evidence of a possible spontaneous remission in certain cases [19]. Up to now, i.e. an almost 4-year follow-up, recurrence has not occurred. Careful long-term follow-up of the patient allowed us to unambiguously exclude other causes of painful ophthalmoplegia, and confirmed the initial diagnosis of Tolosa-Hunt syndrome.

The clinical course of Tolosa-Hunt syndrome in our patient confirms the possibility of bilateral infiltration of the supraorbital fissures and cavernous sinuses. Therefore, although criteria of the syndrome classify unilateral lesions as typical, we believe that the differential diagnosis in patients with bilateral, painful ophthalmoplegia should include Tolosa-Hunt syndrome.

Significant spontaneous regression of the infiltrate of the pituitary and its surrounding (cavernous sinuses and superior orbital fissures) indicates an inflammatory process, and excludes the initial suspicion of a macroadenoma of the gland.
